# Supplementary material for: PUMILIO proteins promote colorectal cancer growth via suppressing p21
Source: Nat Commun. 2022 Mar 25;13:1627. doi: 10.1038/s41467-022-29309-1 (PMC8956581; doi:10.1038/s41467-022-29309-1)
Supplement: Supplementary file 1 — Supplementary Information [file 41467_2022_29309_MOESM1_ESM.pdf]

Supplementary Information

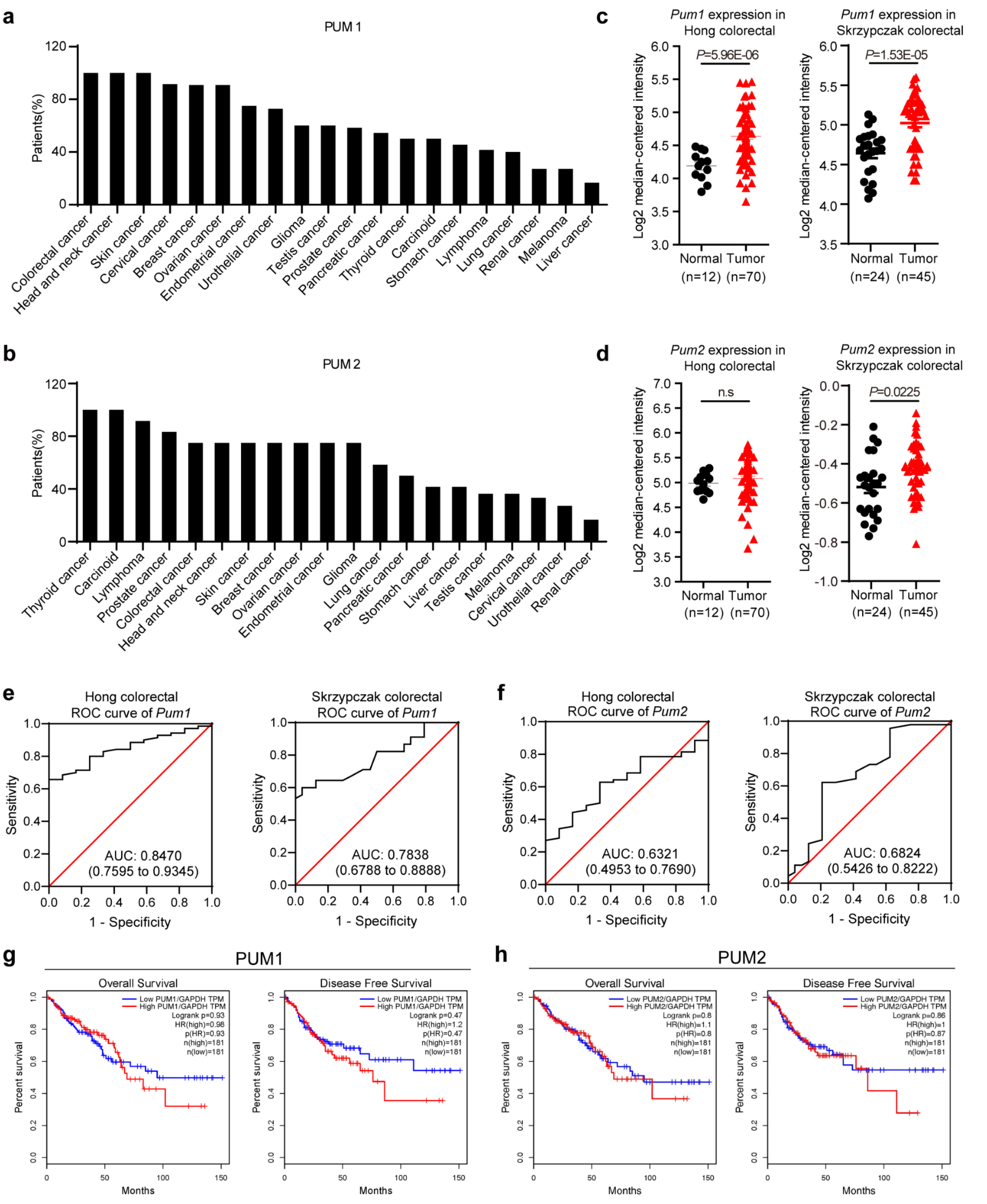

Supplementary Figure 1. PUM1 is markedly overexpressed in colorectal cancer.

a, **b** Protein levels of PUM1 (**a**) or PUM2 (**b**) in different types of cancer using public database (The Human Protein Atlas). “Patients %” indicates the percentage of patients with high and medium protein expression levels. **c**, **d** Expression of *Pum1* (**c**) or *Pum2* (**d**) in human Hong colorectal (n = 12 for normal and n = 70 for cancer group) and Skrzypczak colorectal (n = 24 for normal and n = 45 for cancer group) cancer clinical specimens using the TCGA mRNA

HiSeq expression array data. **e**, **f** ROC curve analysis for colorectal cancer diagnosis. AUC estimation for *Pum1* (**e**) or *Pum2* (**f**) in human colorectal cancer clinical specimens. **g**, **h** Clinical overall survival and disease-free survival of colorectal cancer patients with different levels of PUM1 (**g**) and PUM2 (**h**), in which high and low TPM are defined as the upper and lower 50% PUM1/GAPDH TPM values of the patients, respectively.

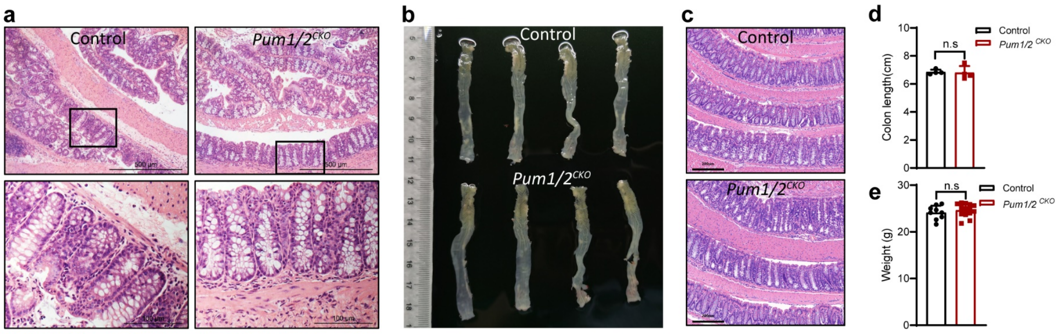

Supplementary Figure 2. The morphology of the small intestine of *Pum1/2*^{CKO} mice is similar to that of the control mice.

a H&E staining of colon tumor in control (*Pum1*$^{flox/flox}$*::Pum2*$^{flox/flox}$) and *Pum1/2*^{CKO} (*Lgr5*^{cre}*::Pum1*$^{flox/flox}$*::Pum2*$^{flox/flox}$) mice in AOM / DSS model. **b** The colon and rectum of control and *Pum1/2*^{CKO} mice in the absence of AOM/DSS. **c** Representative micrographs of H&E staining of colon in control and *Pum1/2*^{CKO} mice in the absence of AOM/DSS. **d** The colon length of control and *Pum1/2*^{CKO} mice without AOM/DSS treatment (n = 4). Error bars represent SD. **e** The weight of control (n = 9) and *Pum1/2*^{CKO} mice (n = 16) without AOM/DSS treatment. Error bars represent SD.

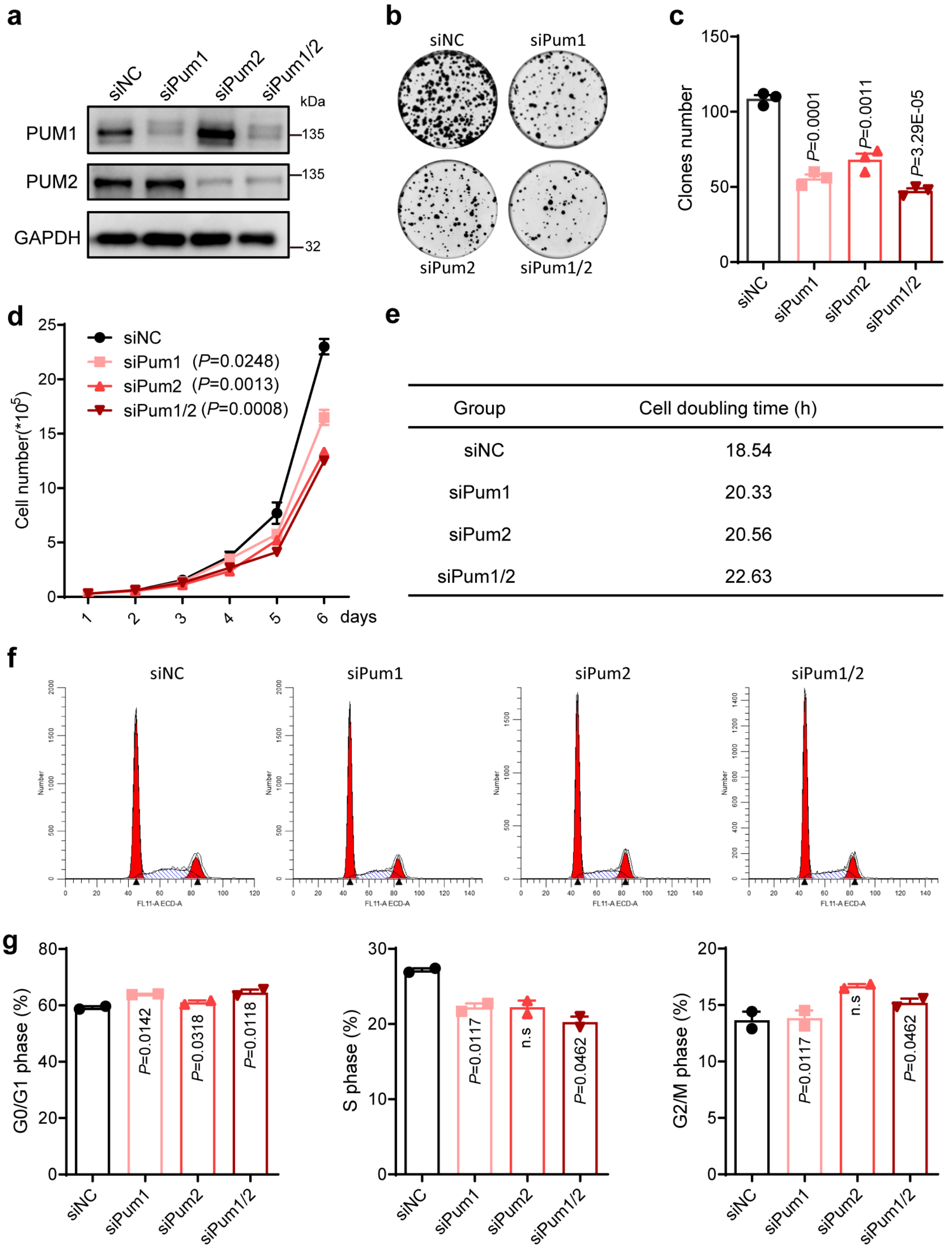

Supplementary Figure 3. Knockdown of *Pumilio* impairs colorectal cancer cells growth.

a Detection of knockdown efficiency of PUM1 and PUM2 by western blot. GAPDH was used as a loading control. **b** Colony formation assay of HCT116 cells transfected with siNC (100 nM), siPum1 (100 nM), siPum2 (100 nM), or siPum1/2 (100 nM). Colony numbers were counted after 10 days. **c** Quantification of clones in (**b**) (n = 3). Error bars represent SD. **d** Growth curves of HCT116 cells transfected with siNC (100 nM), siPum1 (100 nM), siPum2 (100 nM) or siPum1/2 (100 nM) (n = 3). Error bars represent SD. **e** Cell doubling time is calculated by the data in (**d**). **f** Cell cycle of HCT116 cells is determined by PI staining and

cytometry analysis. **g** Quantification of cell cycle by ModFit software (n = 2). Error bars represent SD.

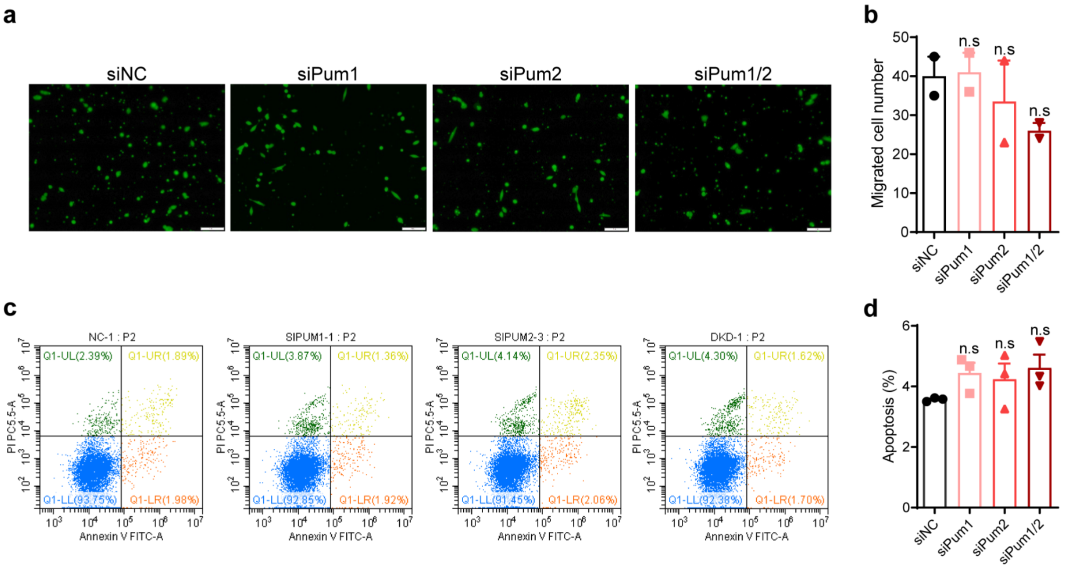

Supplementary Figure 4. Knockdown of *Pum1* and/or *Pum2* has no significant effect on colorectal cancer cells migration and apoptosis.

a Transwell assay of HCT116 cells transfected with siNC (100 nM), siPum1 (100 nM), siPum2 (100 nM), and siPum1/2 (100 nM), respectively. Scale bar = 100 μm. **b** Quantification of (**a**) (n = 2). Error bars represent SD. n.s: not significant. **c** Flow cytometry analysis of cell apoptosis in indicated HCT116 cells using Annexin V and 7AAD. **d** Quantification of (**c**) (n = 3). Error bars represent SD. n.s: not significant.

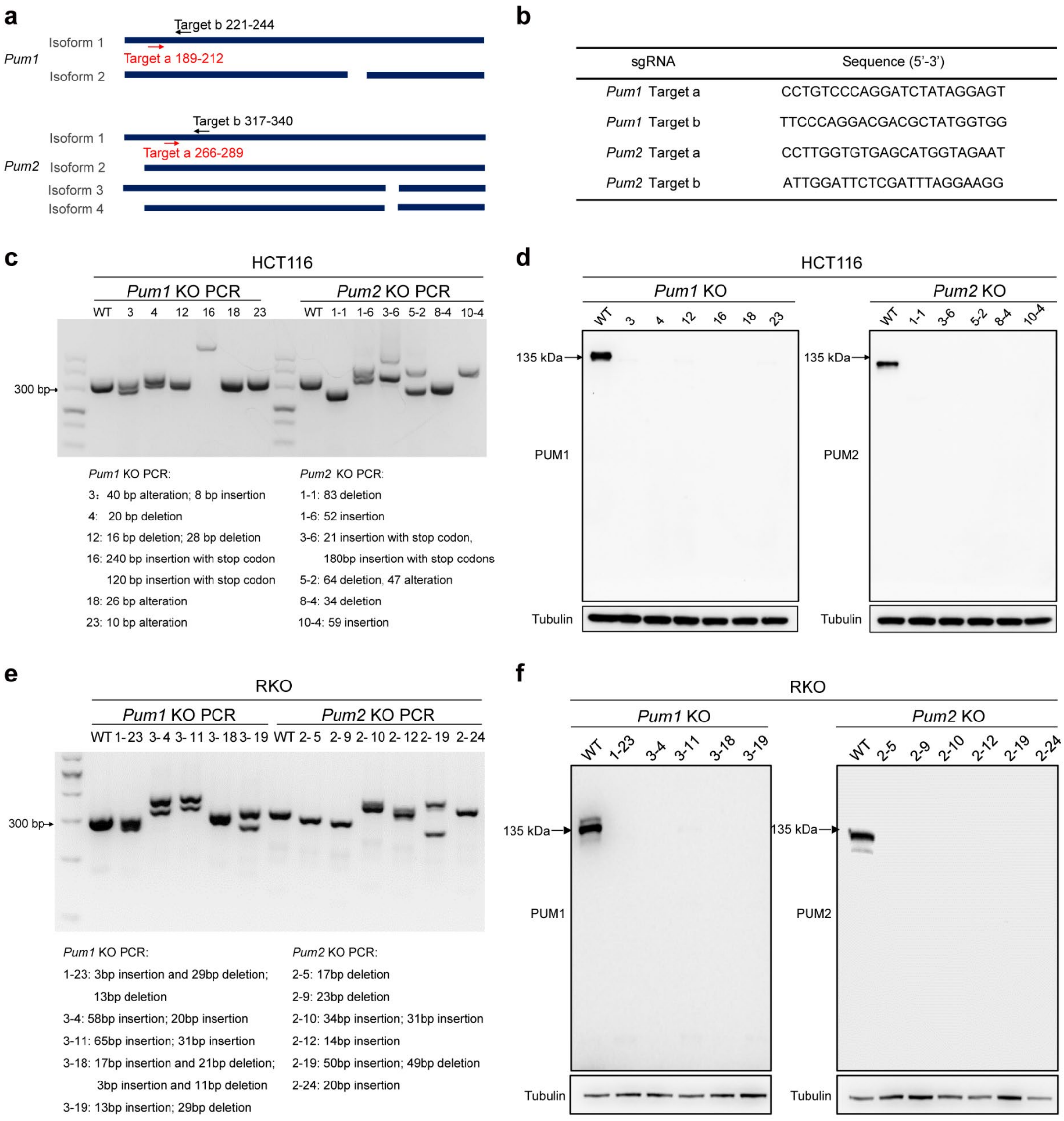

Supplementary Figure 5. Design of sgRNA for *Pumilio* gene knockout.

a Designed sgRNAs target all isoforms of *Pum1* and *Pum2*. **b** Sequence of sgRNAs, designed by an online program: <website http://crispr.mit.edu/>. **c**-**f** *Pum1*$^{-/-}$ and *Pum2*$^{-/-}$ HCT116 (**c**) or RKO (**e**) cell lines are validated by genome PCR and gene mutations are shown below. Validation of *Pum1*$^{-/-}$ and *Pum2*$^{-/-}$ HTC116 (**d**) or RKO (**f**) cell lines by western blotting. No protein truncation can be observed. Tubulin was used as a loading control.

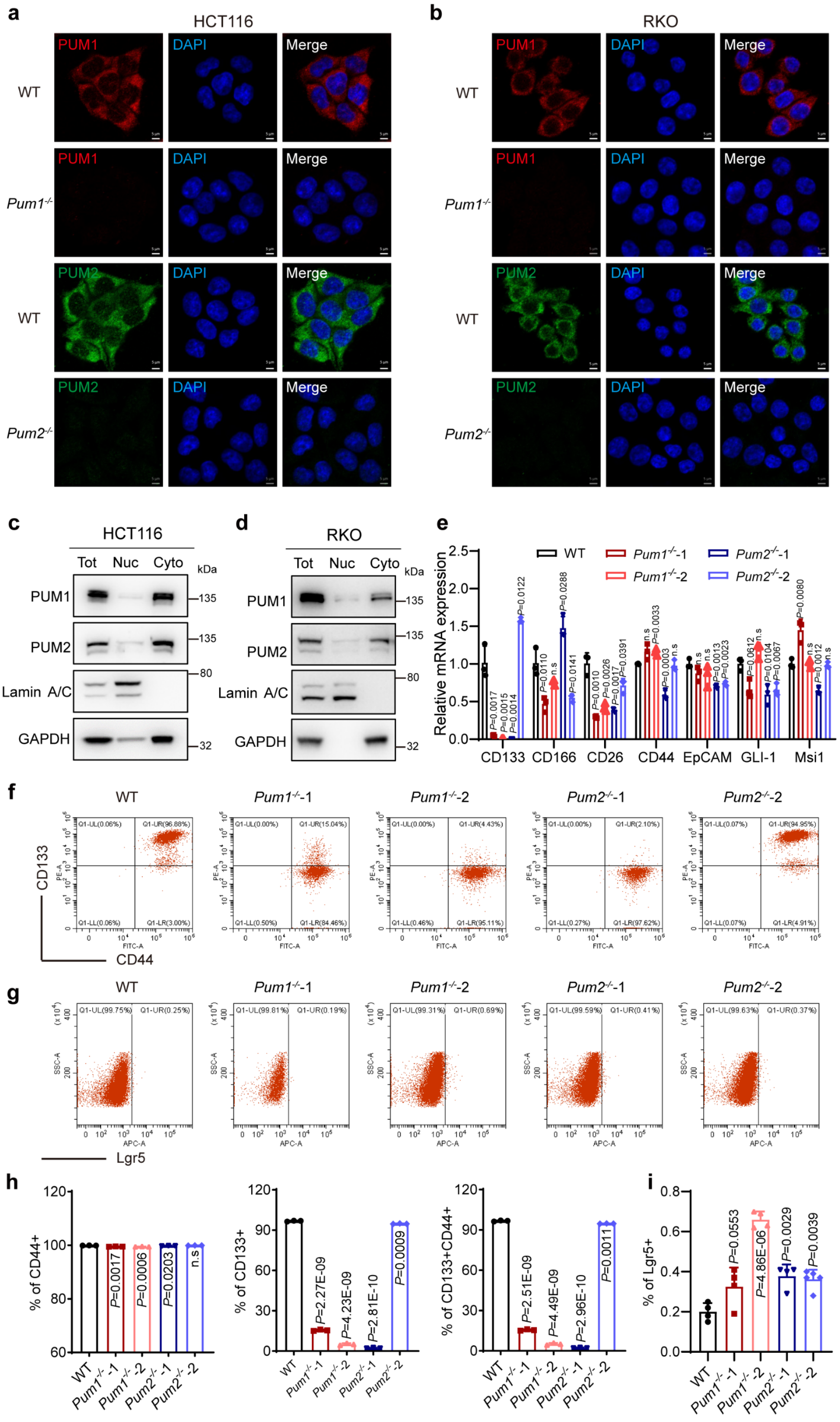

Supplementary Figure 6. PUM1 and PUM2, localized cytoplasmically, are depleted in *Pum1*$^{-/-}$ and *Pum2*$^{-/-}$ HCT116 cells, which affects cancer stem cell-related properties of HCT116 and RKO cells.

a, **b** Immunofluorescence staining of PUM1 (red) and PUM2 (green) proteins in WT, *Pum1*$^{-/-}$, and *Pum2*$^{-/-}$ HCT116 (**a**) or RKO (**b**) cells. **c**, **d** Distribution of PUM1 and PUM2 in the cytoplasm versus the nucleus in HCT116 (**c**) or RKO (**d**) cells, as indicated by subcellular fractionation and Western blotting analysis, in which GAPDH and Lamin A/C were used as cytoplasmic and nuclear markers, respectively. **e** RT-qPCR of the expression of putative cancer stem cell markers mRNAs in HCT116 WT, *Pum1*$^{-/-}$-1, *Pum1*$^{-/-}$-2, *Pum2*$^{-/-}$-1 and *Pum2*$^{-/-}$-2 cells (n = 3). Error bars represent SEM. **f** CD133^{+}CD44^{+} subpopulations were drastically reduced in Pum1 or Pum2-depleted HCT116 cells by FACS analyses. **g** Lgr5^{+} subpopulations were detected in Pum1 or Pum2-depleted HCT116 cells by FACS analyses. **h** Bar graph showing the percentage of CD44 and/or CD133 positive cells in HCT116 WT, *Pum1*$^{-/-}$-1, *Pum1*$^{-/-}$-2, *Pum2*$^{-/-}$-1 and *Pum2*$^{-/-}$-2 cells (n = 3). Error bars represent SD. **i** Bar graph showing the percentage of Lgr5 positive cells in HCT116 WT, *Pum1*$^{-/-}$-1, *Pum1*$^{-/-}$-2, *Pum2*$^{-/-}$-1 and *Pum2*$^{-/-}$-2 cells (n = 4).

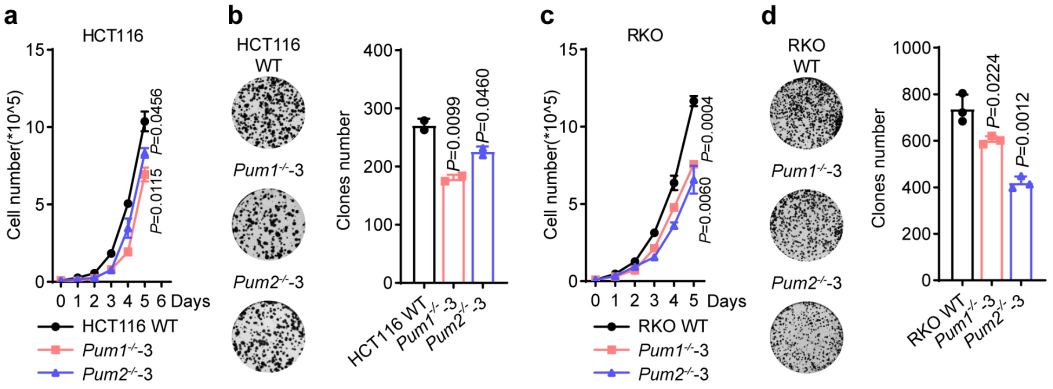

Supplementary Figure 7. Testing more clones of PUM1/2 knockout HCT116 and RKO cell lines.

a Growth curve of WT, *Pum1*$^{-/-}$-3 (#3) and *Pum2*$^{-/-}$-3 (#5-2) in HCT116 cells (n = 3). Error bars represent SD. **b** Colony formation assay of WT, *Pum1*$^{-/-}$-3 (#3) and *Pum2*$^{-/-}$-3 (#5-2) in HCT116 cells (n = 2). Error bars represent SD. **c** Growth curve of WT, *Pum1*$^{-/-}$-3 (#3-11) and *Pum2*$^{-/-}$-3 (#2-9) in RKO cells (n = 3). Error bars represent SD. **d** Colony formation assay of WT, *Pum1*$^{-/-}$-3 (#3-11) and *Pum2*$^{-/-}$-3 (#2-9) in RKO cells (n = 3). Error bars represent SD.

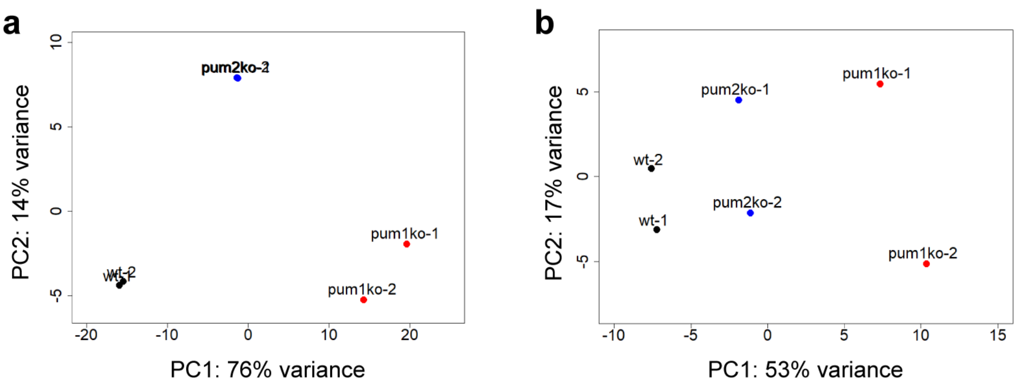

Supplementary Figure 8. Principal component analysis of RNAseq and mass spectrum data.

Principal component analysis of RNAseq (**a**) and mass spectrum (**b**) data based on normalized read counts and normalized peptide abundance, respectively. Samples are plotted according to genotype. Sample-to-sample distances (within and between genotypes) are illustrated for each dataset.

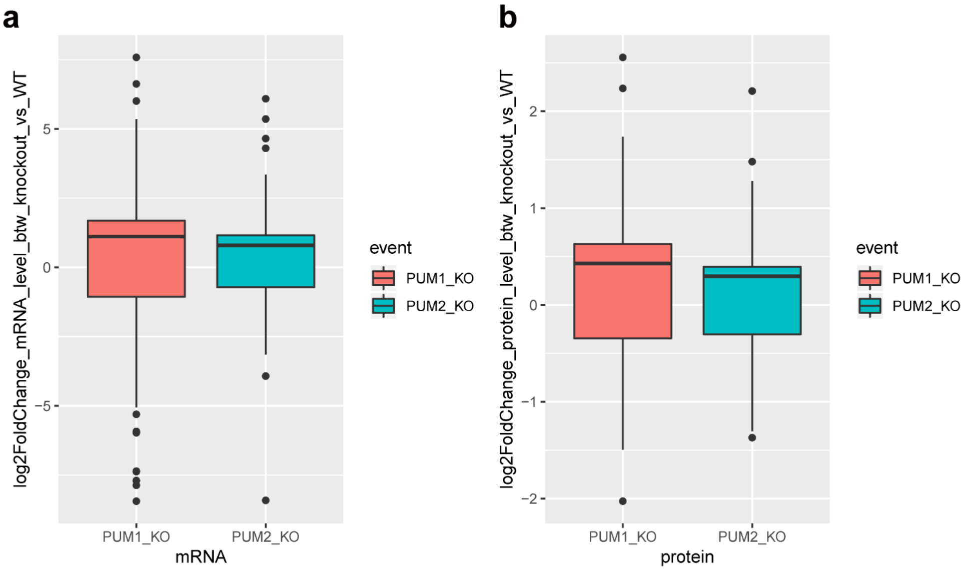

Supplementary Figure 9. Boxplots showing greater changes at mRNA and protein levels in *Pum1*$^{-/-}$ and in *Pum2*$^{-/-}$ cells.

a and **b** show boxplots comparing the log2FoldChange (knockout genotype versus the wild type) between Pum1 knockout (red boxplot) and Pum2 knockout (blue boxplot) (n represent 479 and 532 changed genes in **a** and **b**, respectively). Boxplot represents 5% (lower whisker), 25% (lower box), 50% (median), 75% (upper box), and 95% (upper whisker).

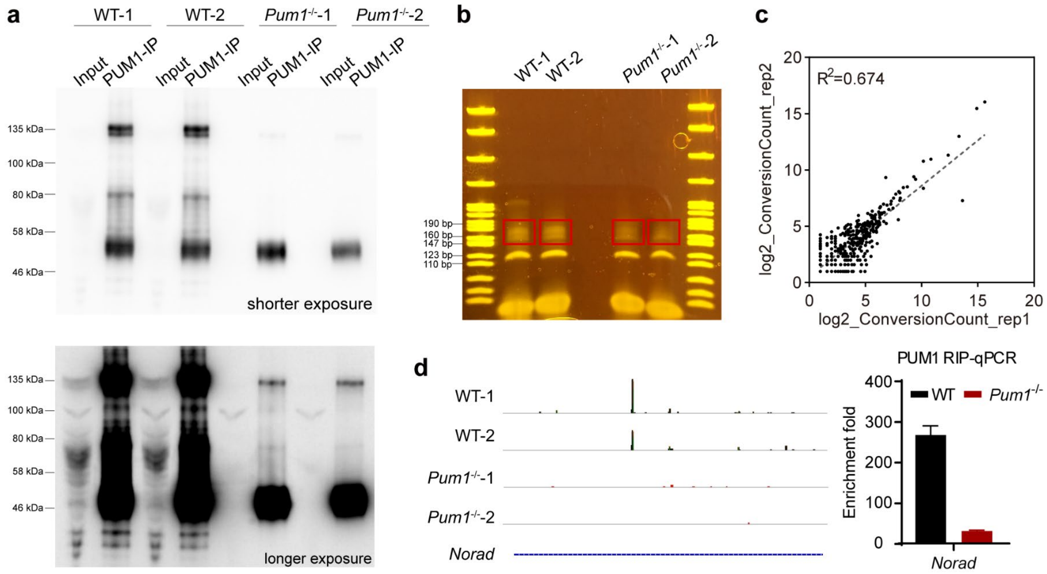

Supplementary Figure 10. PAR-CLIP assay of PUM1 in HCT116 cells.

a PUM1 PAR-CLIP was performed in WT and *Pum1*$^{-/-}$ HCT116 cells, with *Pum1*$^{-/-}$ HCT116 cells as a negative control. Western blot of PUM1-IP validated the IP efficiency for PUM1 antibody. **b** Library preparation for PUM1 PAR-CLIP-seq. The cDNA was separated on the gel and labeled bands were purified for sequencing. **c** Scatter plots comparing transcripts for two biological replicates of PUM1 PAR-CLIP experiments. The square of Spearman's rank correlation value (R^2) for each pair is shown in the top left corner of the respective panel. **d** PUM1 PAR-CLIP peaks on the long non-coding RNA *Norad,* a known target of PUM1, in WT and *Pum1*$^{-/-}$ HCT116 cells (left). PUM1 RIP-qPCR validation of the target *Norad* (right, n = 3). Error bars represent SEM.

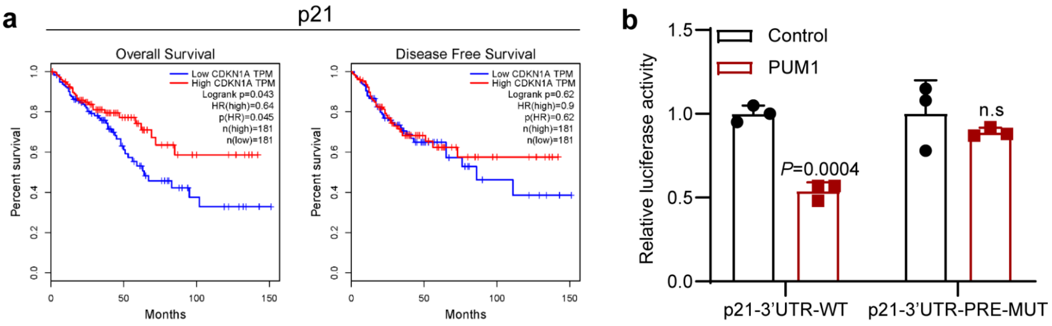

Supplementary Figure 11. Analysis of p21 and PUM1 in colorectal cancer.

a Clinical overall survival and disease free survival of colorectal cancer patients with different levels of p21, in which high and low transcripts per kilobase million (TPM) are defined as the upper and lower 50% TPM values of the patients. **b** The luciferase activity of the p21 3'UTR reporter in HCT116 cells transfected with *Pum1*-overexpression plasmid, 3'UTR of wild type p21 or 3'UTR carrying pumilio response element (PRE) mutation of p21 reporter plasmid for 48 hours (n = 3). Error bars represent SD.

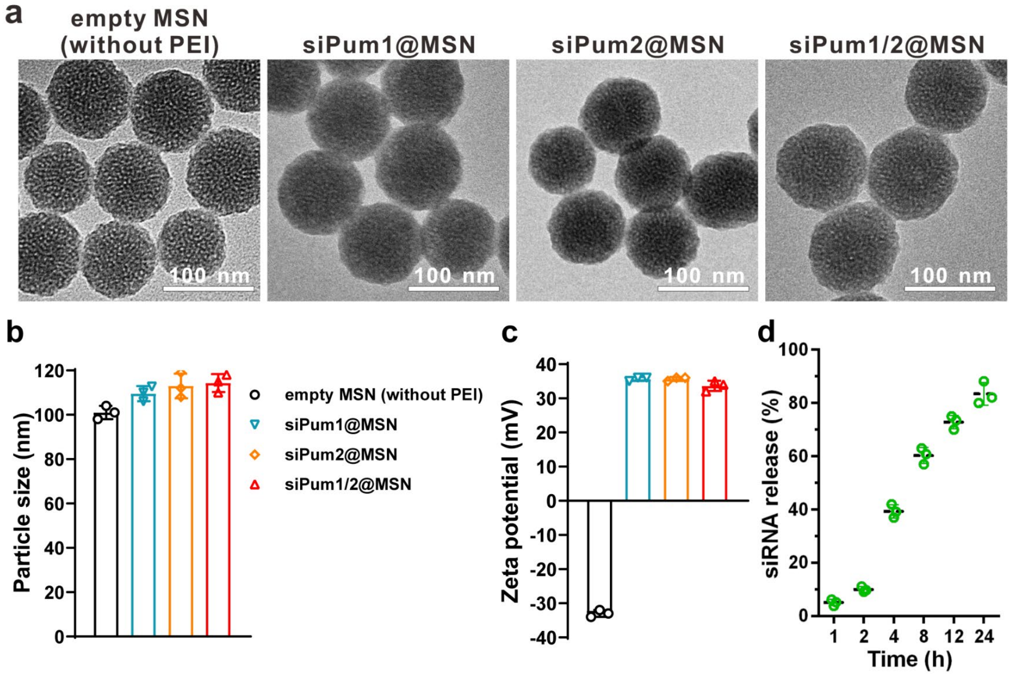

Supplementary Figure 12. Characterization of siRNA-loaded MSN.

a TEM photos of empty MSN, siPum1@MSN, siPum2@MSN, and siPum1/2@MSN. **b** Particle size (n = 3). Error bars represent SD. **c** Zeta potential (n = 3). Error bars represent SD. **d** siRNA release in 5 mM GSH-contained PBS.

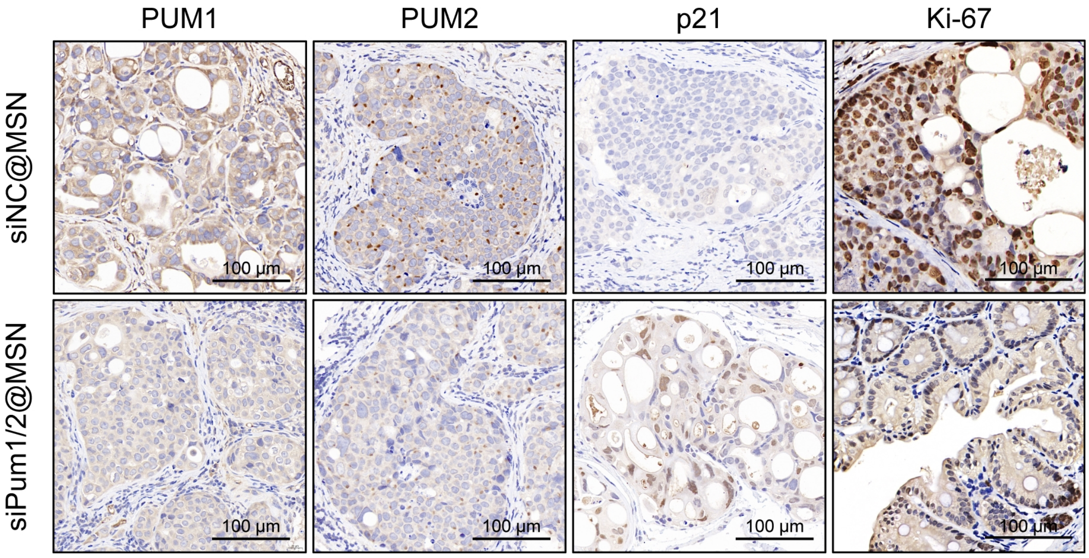

Supplementary Figure 13. Immunochemistry staining of PUM1, PUM2, p21, and Ki-67 in siNC@MSN and siPum1/2@MSN groups.

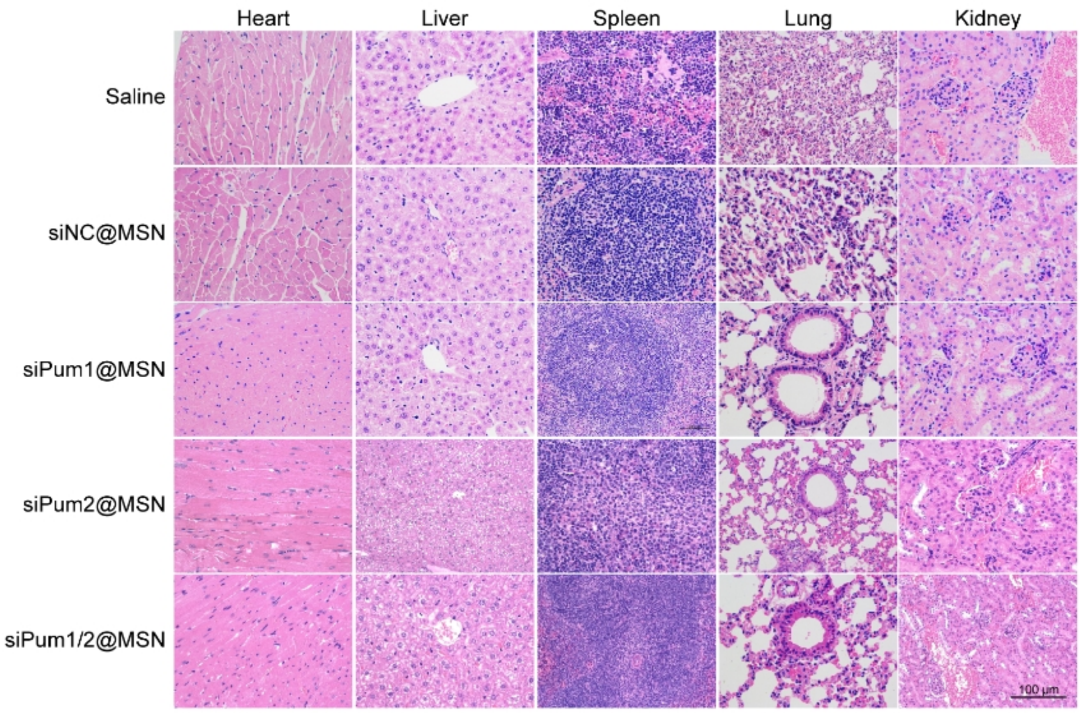

Supplementary Figure 14. Organ toxicology testing of nanoparticle-mediated PUM1 and PUM2 knock- down in the orthotopic colorectal tumor bearing mice.

Three mice from each group were sacrificed on day 11 (2 d after the last injection of the nanoparticles). The heart, liver, spleen, lung, and kidney were collected for H&E histological assay for toxicity evaluation.

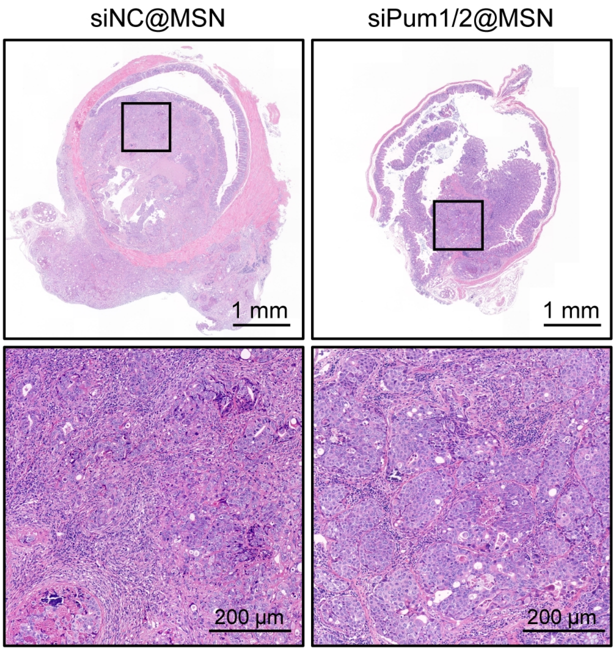

Supplementary Figure 15. Representative H&E staining in siNC@MSN and siPum1/2@MSN group.

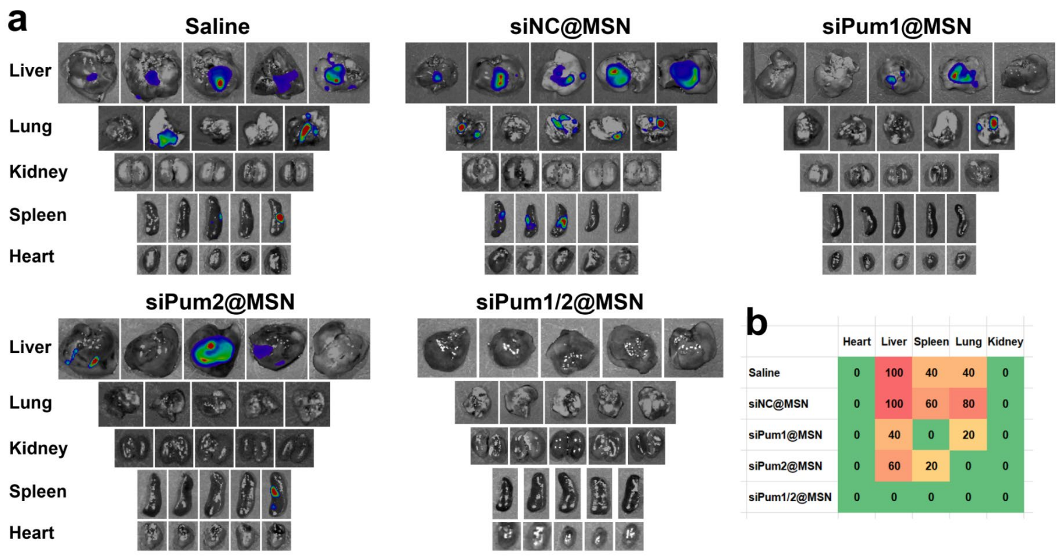

Supplementary Figure 16. siPum1/2@MSN reduced metastatic spread of HCT116-luc tumor cells to other main organs in the orthotopic colorectal tumor bearing mice.

a On last day (Day 30) of the animal study, *ex vivo* bioluminescence imaging was performed to identify micro-metastasis in main organs (heart, liver, spleen, lung, and kidney). **b** A heat map for the metastatic frequency (%) under various treatments.

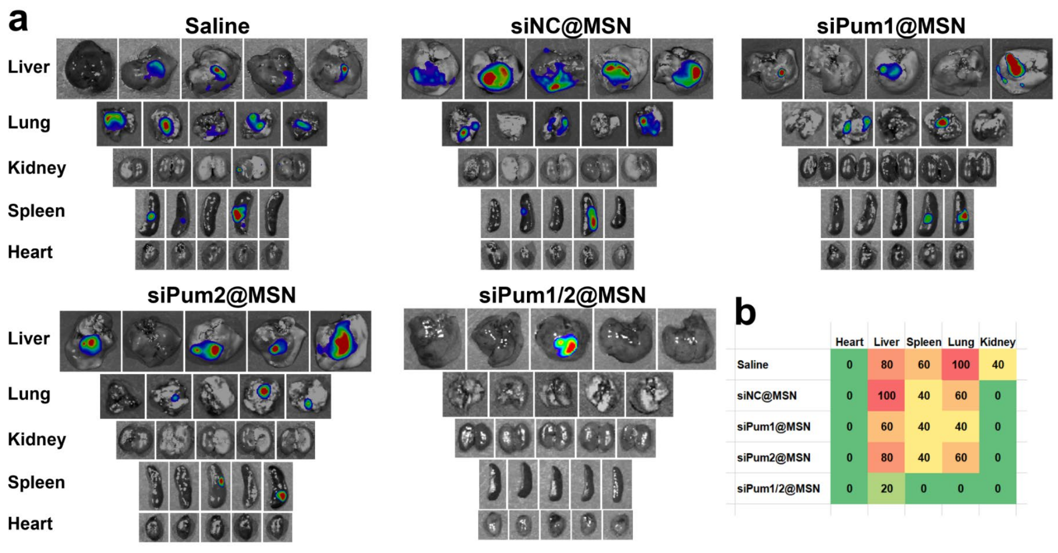

Supplementary Figure 17. siPum1/2@MSN reduced metastatic spread of COLO205-luc tumor cells to other main organs in the orthotopic colorectal tumor bearing mice.

a On last day (Day 30) of the animal study, *ex vivo* bioluminescence imaging was performed to identify micro-metastasis in main organs (heart, liver, spleen, lung, and kidney). **b** A heat map for the metastatic frequency (%) under various treatments.

Supplementary Table 1. Primers used in this paper

Name	Primers 5'-3'	
Pum1 real time PCR	Forward	GGCATGGAGCCTCTTCAGTT
	Reverse	TCAGCGTCCTCTTACTCCCA
Pum2 real time PCR	Forward	TCAGCGTCCTCTTACTCCCA
	Reverse	CCAACCACTAAGGCACCAGT
p21 real time PCR	Forward	TGTCCGTCAGAACCCATGC
	Reverse	AAAGTCGAAGTTCCATCGCTC
Areg real time PCR	Forward	GAGCCGACTATGACTACTCAGA
	Reverse	TCACTTTCCGTCTTGTTTTGGG
Rps9 real time PCR	Forward	GAAATCTCGTCTCGACCAAGAG
	Reverse	GGTCCTTCTCATCAAGCGTCA
Dkk1 real time PCR	Forward	CCTTGAACTCGGTTCTCAATTCC
	Reverse	CAATGGTCTGGTACTTATTCCCG
Nptn real time PCR	Forward	GAGGTCATTATTCGAGACAGCC
	Reverse	TTGATCCTGTACTCCATGTTGC
Slc2a1 real time PCR	Forward	GGCCAAGAGTGTGCTAAAGAA
	Reverse	ACAGCGTTGATGCCAGACAG
Cep97 real time PCR	Forward	CCGGAGAAGGATCAGTGGTCA
	Reverse	CCACACCCATCATCCGAAC
Ccnb1 real time PCR	Forward	AACTTTCGCCTGAGCCTATTTT
	Reverse	TTGGTCTGACTGCTTGCTCTT
Plk1 real time PCR	Forward	CACCAGCACGTCGTAGGATTC
	Reverse	CCGTAGGTAGTATCGGGCCTC
Kpna2 real time PCR	Forward	ACATAATCCGGGCTGGTTTGA
	Reverse	GGGAGATGCCAACAGAGAAATG
Bub3 real time PCR	Forward	GGACCCATGATGCCCCTATC
	Reverse	CCCAGCATTACAAGGAGTTCTG
Mtmr10 real time PCR	Forward	GCAAATTGTCACAGTAAACGACC
	Reverse	TGGCTGGGAATAATGAGCTATTG
Upf1 real time PCR	Forward	ACCTATTACACGAAGGACCTCC
	Reverse	ACGTCCGTTGCAGAACCAC
Hsp90aa1 real time PCR	Forward	GCTTGACCAATGACTGGGAAG
	Reverse	AGCTCCTCACAGTTATCCATGA
Coq10b real time PCR	Forward	GGACTGGTCATACGGCCTTG
	Reverse	ACCACAGGAAGCTAAATATCTGC
Hsp90ab1 real time PCR	Forward	CGAAGTTGGACAGTGGTAAAGAG
	Reverse	TGCCCAATCATGGAGATGTCT
Myc real time PCR	Forward	GGCTCCTGGCAAAAGGTCA
	Reverse	CTGCGTAGTTGTGCTGATGT

Norad real time PCR	Forward	AGCGAAGTCCCGAACGACGA
	Reverse	TGGGCATTTCCAACGGGCCAA
CD133 real time PCR	Forward	AGTCGGAAACTGGCAGATAGC
	Reverse	GGTAGTGTTGTACTGGGCCAAT
CD166 real time PCR	Forward	ACTTGACGTACCTCAGAATCTCA
	Reverse	CATCGTCGTACTGCACACTTT
CD26 real time PCR	Forward	GGGTCACATGGTCACCAGTG
	Reverse	TCTGTGTCGTTAAATTGGGCATA
CD44 real time PCR	Forward	CTGCCGCTTTGCAGGTGTA
	Reverse	CATTGTGGGCAAGGTGCTATT
EpCAM real time PCR	Forward	AATCGTCAATGCCAGTGTACTT
	Reverse	TCTCATCGCAGTCAGGATCATAA
GLI-1 real time PCR	Forward	AGCGTGAGCCTGAATCTGTG
	Reverse	CAGCATGTACTGGGCTTTGAA
Msi1 real time PCR	Forward	GGGACTCAGTTGGCAGACTAC
	Reverse	CTGGTCCATGAAAGTGACGAA
si*Pum1*	Forward	UCACAUGGAAACUCUGACCTT
	Reverse	UAUGCAUGACGAUCUUCCGTT
si*Pum2*	Forward	AAGCGCUCAACUACUUCAGTT
	Reverse	UCGUGUUACUGUUAUGUCUTT
p21 PRE^{mut} PCR	Forward	ACGTTCCCCGAGTTCTTCCT
	Reverse	GAGGAAGTAGCTGGCATGAA
Actin real time PCR	Forward	CATGTACGTTGCTATCCAGGC
	Reverse	CTCCTTAATGTCACGCACGAT
Gapdh real time PCR	Forward	TCTTCTCGTGCAGTGCTAGC
	Reverse	TGGTTTCATGACAAGGTAGGGC
Pum1-CDS	Forward	CTAGCTAGCATGAGCGTTGCATGTGTCTTGAAGAGAAAAGC
	Reverse	CCGCTCGAGTCAGATGATACCATTAGGGGGGCCACAGATGG
Pum1 KO PCR	Forward	CAGTGCTTTGGCAGGACTCTTTCAG
	Reverse	CTGATGTTCTGCATGAATGTTATCCCC
Pum2 KO PCR	Forward	CCATTCAATGTCCCAGCCTATTATGG
	Reverse	CAAGTGGCTAAGCAGAAGAATAGC
p21-3' UTR-WT	Forward	AATTCTAGGCGATCGCTCGATCCGCCCACAGGAAGCCT
	Reverse	AAACGAATTCCCGGGCTCGAGAGCACCTGCTGTATATTCAGCAT
p21-3' UTR-PRE-MUT	Forward	ACATCATACCCTGGCCGCCC
	Reverse	TGAGGAGGTGTTTAAATTAAAACAC

Supplementary Table 2. List of unique peptides of PUM1 and PUM2 in wild type, *Pum1*$^{-/-}$ and *Pum2*$^{-/-}$ cells

Protein	Unique Peptides	WT			*Pum1*$^{-/-}$ *or Pum2*$^{-/-}$		
		run1	run2	run3	run1	run2	run3
PUM1	DSAWGTSDHSVSQPIMVQR	1	1	0	0	0	0
	WPTGDNIHAEHQVR	1	1	1	0	0	0
	SASSASSLFSPSSTLFSSSR	1	1	0	0	0	0
	SQDDAMVDYFFQR	1	1	1	0	0	0
	GIFLGDQWR	1	0	0	0	0	0
	AVLIDEVCTMNDGPHSALYTMMK	1	1	1	0	0	0
	MIDVAEPGQR	0	1	0	0	0	0
	SMDELNHDFQALALEGR	0	1	1	0	0	0
	FWETDESSKDGPK	0	0	1	0	0	0
PUM2	ETAWGASHHSMSQPIMVQR	1	1	1	0	0	0
	DAETDGPEKGDQK	1	0	0	0	0	0
	ALLIDEVCCQNDGPHSALYTMMK	1	0	1	0	0	0
	ASPFEEDQNR	0	1	1	0	0	0
