# Supplementary material for: PUMILIO proteins promote colorectal cancer growth via suppressing p21
Source: Nat Commun. 2022 Mar 25;13:1627. doi: 10.1038/s41467-022-29309-1 (PMC8956581; doi:10.1038/s41467-022-29309-1)
Supplement: Supplementary file 3 — Description of Additional Supplementary Files [file 41467_2022_29309_MOESM3_ESM.pdf]

Description of Additional Supplementary Files

File Name: Supplementary Data 1
Description: mRNAs differentially expressed in *Pum1-/-* HCT116 cells

File Name: Supplementary Data 2
Description: mRNAs differentially expressed in *Pum2*$^{-/-}$ HCT116 cells

File Name: Supplementary Data 3
Description: Proteins differentially expressed in *Pum1*$^{-/-}$ HCT116 cells

File Name: Supplementary Data 4
Description: Proteins differentially expressed in *Pum2*$^{-/-}$ HCT116 cells

File Name: Supplementary Data 5
Description: KEGG analysis of mRNAs and proteins changed in *Pum1*$^{-/-}$ and *Pum2*$^{-/-}$ cells

File Name: Supplementary Data 6
Description: Summary of statistics for PUM1 PAR-CLIPs in HCT116 cell lines

File Name: Supplementary Data 7
Description: KEGG analysis of PUM1 PARCLIP
