# Supplementary material for: PUMILIO proteins promote colorectal cancer growth via suppressing p21
Source: Nat Commun. 2022 Mar 25;13:1627. doi: 10.1038/s41467-022-29309-1 (PMC8956581; doi:10.1038/s41467-022-29309-1)
Supplement: Supplementary file 11 — Reporting Summary [file 41467_2022_29309_MOESM11_ESM.pdf]

nature portfolio

Corresponding author(s): Sanhong Liu
Haifan Lin
Chao Fang

Last updated by author(s): Feb 6, 2022

Reporting Summary

Nature Portfolio wishes to improve the reproducibility of the work that we publish. This form provides structure for consistency and transparency in reporting. For further information on Nature Portfolio policies, see our Editorial Policies and the Editorial Policy Checklist.

Statistics

For all statistical analyses, confirm that the following items are present in the figure legend, table legend, main text, or Methods section.

n/a	Confirmed	
☐	☒	The exact sample size (*n*) for each experimental group/condition, given as a discrete number and unit of measurement
☐	☒	A statement on whether measurements were taken from distinct samples or whether the same sample was measured repeatedly
☐	☒	The statistical test(s) used AND whether they are one- or two-sided<br>*Only common tests should be described solely by name; describe more complex techniques in the Methods section.*
☒	☐	A description of all covariates tested
☐	☒	A description of any assumptions or corrections, such as tests of normality and adjustment for multiple comparisons
☐	☒	A full description of the statistical parameters including central tendency (e.g. means) or other basic estimates (e.g. regression coefficient) AND variation (e.g. standard deviation) or associated estimates of uncertainty (e.g. confidence intervals)
☒	☐	For null hypothesis testing, the test statistic (e.g. *F*, *t*, *r*) with confidence intervals, effect sizes, degrees of freedom and *P* value noted<br>*Give P values as exact values whenever suitable.*
☒	☐	For Bayesian analysis, information on the choice of priors and Markov chain Monte Carlo settings
☒	☐	For hierarchical and complex designs, identification of the appropriate level for tests and full reporting of outcomes
☐	☒	Estimates of effect sizes (e.g. Cohen's *d*, Pearson's *r*), indicating how they were calculated

Our web collection on statistics for biologists contains articles on many of the points above.

Software and code

Policy information about availability of computer code

Data collection	All software is publicly or commercially available.<br>Western blot images were acquired using ChemiDOC Touch Imaging System (BIO-RAD).<br>Fluorescence microscopic images were acquired using Zeiss LSM 710 confocal microscope (Zeiss).<br>Flow cytometry data were collected using CytoFLEX (Beckman Coulter).<br>Mass spectrum data were collected using Nexera X2 LC-30AD HPLC.<br>The tumor burden was monitored weekly using bioluminescence imaging (IVIS spectrum CT).
Data analysis	Bioinformatic analyses (PUM1 PAR-CLIP) were performed using TrimGalore (version 0.4.4_dev), bowtie (version 1.2.1.1), samtools (version 1.4.1), PARalyzer (version 1.5), bedtools (version 2.22.0).<br>Mass spectrum data analyses were performed using Proteome discover (version 2.2).<br>Fluorescence microscopic images analyses were performed using ZEN (version 2012).<br>Flow cytometry data analyses were performed using CytoExpert (version 2.0) and Modifit (LT 5.0).<br>Statistical analysis was performed using GraphPad Prism 8 software.<br>A custom script used to analyse the PAR-CLIP data is available in the Zenodo repository under DOI: 10.5281/zenodo.5981256.

For manuscripts utilizing custom algorithms or software that are central to the research but not yet described in published literature, software must be made available to editors and reviewers. We strongly encourage code deposition in a community repository (e.g. GitHub). See the Nature Portfolio guidelines for submitting code & software for further information.

Data

Policy information about availability of data

All manuscripts must include a data availability statement. This statement should provide the following information, where applicable:

- Accession codes, unique identifiers, or web links for publicly available datasets
- A description of any restrictions on data availability
- For clinical datasets or third party data, please ensure that the statement adheres to our policy

The accession number for the RNA-seq and PAR-CLIP data reported in this paper is GEO: PRJNA648706. The mass spectrometry proteomics data have been deposited to the ProteomeXchange Consortium (http://proteomecentral.proteomexchange.org) via the iProX partner repository with the dataset identifier PXD027513. The human hg19 genome are available from the ensembl.org. The Uniprot_Human data are available from uniprot.org. Source data are provided with this paper.

Field-specific reporting

Please select the one below that is the best fit for your research. If you are not sure, read the appropriate sections before making your selection.

☒ Life sciences ☐ Behavioural & social sciences ☐ Ecological, evolutionary & environmental sciences

For a reference copy of the document with all sections, see nature.com/documents/nr-reporting-summary-flat.pdf

Life sciences study design

All studies must disclose on these points even when the disclosure is negative.

Sample size	No sample size calculation were performed.<br>Sample size was determined based on previously published studies(ref. 13, 18, 24, 44) and on experience.
Data exclusions	Data were only excluded for failed experiments resulting from technical issues, such as no tumor-bearing mice in figure 6D and H.
Replication	Biological replicates in each experiments are defined in the figure legends. Results were at least performed 2 independent times and showed similar results.
Randomization	For tumorigenesis assay and antitumor therapy in orthotopic colorectal tumor model, Mice were randomly allocated into different experimental groups before being injected with the cell lines of interest.<br>For AOM/DSS model, Mice were randomly allocated into different cages before being treated with AOM and DSS.<br>For experiments involving cellular and biological studies, no randomization is applied because the study was based on molecular and cellular biology, similar results were obtained from at least 2 independent times with 2 independent people instead.
Blinding	Mice work including AOM/DSS model, antitumor therapy in orthotopic colorectal tumor model and tumorigenesis assay were blinded. For other experiments, the results were quantified and appropriate statistical tests were performed to evaluate difference and statistical significance, similar results were obtained from at least 2 independent times with 2 independent people.

Reporting for specific materials, systems and methods

We require information from authors about some types of materials, experimental systems and methods used in many studies. Here, indicate whether each material, system or method listed is relevant to your study. If you are not sure if a list item applies to your research, read the appropriate section before selecting a response.

Materials & experimental systems

n/a	Involved in the study	
☐	☒	Antibodies
☐	☒	Eukaryotic cell lines
☒	☐	Palaeontology and archaeology
☐	☒	Animals and other organisms
☒	☐	Human research participants
☒	☐	Clinical data
☒	☐	Dual use research of concern

Methods

n/a	Involved in the study	
☒	☐	ChIP-seq
☐	☒	Flow cytometry
☒	☐	MRI-based neuroimaging

Antibodies

Antibodies used	For western blot, the following antibodies were used: Recombinant Anti-Pumilio 1 antibody [EPR3795](1:1000, Abcam, Cat#:

Antibodies used

ab92545), Recombinant Anti-Pumilio 2 antibody [EPR3813] (1:1000, Abcam, Cat#: ab92390), Recombinant Anti-Lamin A + Lamin C antibody [EPR4100] - Nuclear Envelope Marker (1:1000, Abcam, Cat#: ab108595), p21 Waf1/Cip1 (12D1) Rabbit mAb (1:1000, cell signaling technology, Cat#: 2947S), α-Tubulin (11H10) Rabbit mAb (1:1000, cell signaling technology, Cat#: 2125S), GAPDH (14C10) Rabbit mAb (1:1000, cell signaling technology, Cat#: 2118S), β-Actin (13E5) Rabbit mAb (1:1000, cell signaling technology, Cat#: 4970S) and HRP-conjugated Affinipure Goat Anti-Rabbit IgG(H+L) (1:5000, proteintech, Cat#: SA00001-2).

For IF or/and IHC, the following antibodies were used: Recombinant Anti-Pumilio 1 antibody [EPR3795](1:200, Abcam, Cat#: ab92545), Recombinant Anti-Pumilio 2 antibody [EPR3813] (1:200, Abcam, Cat#: ab92390), p21 Waf1/Cip1 (12D1) Rabbit mAb (1:200, cell signaling technology, Cat#: 2947S), Anti-Ki67 antibody (1:200, Santa Cruz Biotechnology Inc, Cat#: sc-7846), Goat anti-Rabbit IgG (H+L) Highly Cross-Adsorbed Secondary Antibody, Alexa Fluor Plus 555 (1:1000, invitrogen, Cat#: A32732) and HRP-conjugated Affinipure Goat Anti-Rabbit IgG(H+L) (1:1000, proteintech, Cat#: SA00001-2)

For PAR-CLIP or/and RIP, the following antibodies were used: Recombinant Anti-Pumilio 1 antibody [EPR3795] (0.25mg/ml, Abcam, Cat#: ab92545) and Recombinant Anti-Pumilio 2 antibody [EPR3813] (0.25mg/ml, Abcam, Cat#: ab92390)

Validation

Recombinant Anti-Pumilio 1 antibody [EPR3795](Abcam, Cat#: ab92545)
Species reactivity: Mouse, Rat, Human
Application validated by manufacturer: ICC/IF, WB, IHC-P, Flow Cyt (Intra)
Application validated by published papers: IP, CLIP, RIP (have been cited > 10 times, for example, DOI: 10.1101/gad.298752.117, DOI: 10.1073/pnas.1916471117)

Recombinant Anti-Pumilio 2 antibody [EPR3813] (Abcam, Cat#: ab92390)
Species reactivity: Mouse, Rat, Human
Application validated by manufacturer: Flow Cyt (Intra), WB, IP, ICC/IF
Application validated by published papers: CLIP, RIP (have been cited > 10 times, for example, DOI: 10.1101/gad.298752.117, DOI: 10.1073/pnas.1916471117)

Recombinant Anti-Lamin A + Lamin C antibody [EPR4100] - Nuclear Envelope Marker (Abcam, Cat#: ab108595)
Species reactivity: human
Application validated by manufacturer: Flow Cyt (Intra), ICC/IF, WB, IP, IHC-P

p21 Waf1/Cip1 (12D1) Rabbit mAb (cell signaling technology, Cat#: 2947S)
Species reactivity: Human, Monkey
Application validated by manufacturer: WB, IP, IHC, IF, F

α-Tubulin (11H10) Rabbit mAb (cell signaling technology, Cat#: 2125S)
Species reactivity: Human, Mouse, Rat, Monkey, D. melanogaster, Zebrafish, Bovine, Pig
Application validated by manufacturer: WB, IHC, IF, F

GAPDH (14C10) Rabbit mAb (cell signaling technology, Cat#: 2118S)
Species reactivity: Human, Mouse, Rat, Monkey, Bovine, Pig
Application validated by manufacturer: WB, IHC, IF, F

β-Actin (13E5) Rabbit mAb (cell signaling technology, Cat#: 4970S)
Species reactivity: Human, Mouse, Rat, Monkey, Bovine, Pig
Application validated by manufacturer: WB, IHC, IF, F

Anti-Ki67 antibody (M19)(Santa Cruz Biotechnology Inc, Cat#: sc-7846)
Species reactivity: Mouse, Human, Rat
Application validated by manufacturer: ELISA, FC/FACS, IF, IP, WB

Eukaryotic cell lines

Policy information about cell lines

Cell line source(s)

HCT116, RKO, COLO205, LOVO, SW480, SW620, HT29 cells were cultured according to the culture methods of ATCC.
Colon normal immortalized epithelial cell line NCM460 was obtained from In Cell (San Antonio, TX) and cultured according to the method of manufacturer.

Authentication

All cells were authenticated using STR.

Mycoplasma contamination

All cell lines tested negative for Mycoplasma Contamination.

Commonly misidentified lines
(See ICLAC register)

None of the cell lines used in this study are commonly misidentified.

Animals and other organisms

Policy information about studies involving animals; ARRIVE guidelines recommended for reporting animal research

Laboratory animals

For tumorigenesis assay, 4 week old BALB/c nude mice (male) were purchased from Shanghai Lingchang Biotechnology Co., Ltd (Shanghai, China).
For AOM/DSS model, 6-8 weeks old (male) C57BL/6J Lgr5cre::Pum1flox/flox::Pum2flox/flox and Pum1flox/flox::Pum2flox/flox mice were used.

	For antitumor Therapy in Orthotopic Colorectal Tumor Model, 4-5 weeks old (female) BALB/c nude mice were purchased from Shanghai Lingchang Biotechnology Co., Ltd (Shanghai, China).<br>Mice were housed pathogen-free and ventilated cages, and allowed free access to food and autoclaved water (add 2%-2.5% DSS when needed) ad libitum in a 12h light/dark cycle, with room temperature at 21±2°C and humidity between 45 and 65%.
Wild animals	This study did not involve wild animals.
Field-collected samples	This study did not involve field-collected samples.
Ethics oversight	All animal experiments were performed in compliance with the Guide for the Care and Use of Laboratory Animals and approved by the Institutional Biomedical Research Ethics Committee of the ShanghaiTech University or Shanghai Jiao Tong University School of Medicine (SJTU-SM).

Note that full information on the approval of the study protocol must also be provided in the manuscript.

Flow Cytometry

Plots

Confirm that:

☒ The axis labels state the marker and fluorochrome used (e.g. CD4-FITC).

☒ The axis scales are clearly visible. Include numbers along axes only for bottom left plot of group (a 'group' is an analysis of identical markers).

☒ All plots are contour plots with outliers or pseudocolor plots.

☒ A numerical value for number of cells or percentage (with statistics) is provided.

Methodology

Sample preparation	For cell cycle analysis, Cells within 2-5×106 were collected, then wash the cells twice with 1 ml cold PBS, each time centrifuged at 400 g for 5 minutes at 4℃. Discard most of the supernatant and gently bounce the cell sediment evenly, so that the volume of cell suspension is about 50-100ul. Use a whirlpool oscillator to keep the cells in suspension, slowly drop by drop add 1 ml of ice-cold 75% ethanol (pre-cooled at -20℃). Fixed overnight at 4℃ or for 4 hours at -20℃. Cells were collected by centrifugation at 1000 g for 5 min at 4℃ before analysis. Wash cells once with 1 ml cold PBS, resuspend cells with 400 µl cold PBS. Add 400 µl PI Staining Solution/RNase solution, gently mix and incubate for 30-60 minutes in the dark at 4℃ then analyzed by flow cytometry.<br>For cell apoptosis analysis, cells were stained with the FITC Annexin V apoptosis detection kit (556547, BD Biosciences) or PE Annexin V apoptosis detection Kit (559763, BD Biosciences) according to the manufacturer protocol and analyzed early- and late-stage apoptosis by flow cytometry.
Instrument	CytoFLEX, Beckman Coulter
Software	CytExpert, ModFit LT
Cell population abundance	HCT116 fluorescence minus one (FMO) controls were used as negative controls and the proportion of positive cells is 0%, The proportion of AnnxinV+PI- and AnnxinV+PI- cells were 3.57%, 4.44%, 4.24% and 4.61% in hct116 transfected with siNC (100 nM), siPum1 (100 nM), siPum2 (100 nM), and siPum1/2 (100 nM), respectively. The proportion of CD44 and CD133 positive cells were 96.73%, 15.54%, 5.13%, 2.24% and 94.78% in hct116 wt, Pum1-/--1, Pum1-/--2, Pum2-/--1 and Pum2-/--2 cells, respectively. The proportion of Lgr5 positive cells were 0.2%, 0.325%, 0.66%, 0.3775% and 0.36% in hct116 wt, Pum1-/--1, Pum1-/--2, Pum2-/--1 and Pum2-/--2 cells, respectively.
Gating strategy	Cells were defined based on FSC-A and SSC-A profile. singlets were based on the pattern of SSC-A vs SSC-H. FMOs were used to determine the threshold between positive and negative controls.

☒ Tick this box to confirm that a figure exemplifying the gating strategy is provided in the Supplementary Information.
